# ﻿Morphology and molecular phylogeny of *Pleurosigmapacificum* sp. nov. (Pleurosigmataceae), a new tropical pelagic species from the Western Pacific Ocean

**DOI:** 10.3897/phytokeys.227.103890

**Published:** 2023-06-02

**Authors:** Fei-Chao Du, Yu-Hang Li, Kui-Dong Xu

**Affiliations:** 1 Laboratory of Marine Organism Taxonomy and Phylogeny, Qingdao Key Laboratory of Marine Biodiversity and Conservation, Shandong Province Key Laboratory of Experimental Marine Biology, Institute of Oceanology, Chinese Academy of Sciences, Qingdao 266071, China Institute of Oceanology, Chinese Academy of Sciences Qingdao China; 2 University of Chinese Academy of Sciences, Beijing 100049, China University of Chinese Academy of Sciences Beijing China; 3 Laboratory for Marine Biology and Biotechnology, Pilot National Laboratory for Marine Science and Technology (Qingdao), Qingdao 266237, China Pilot National Laboratory for Marine Science and Technology Qingdao China

**Keywords:** Marine diatoms, morphology, new species, phylogeny, *Pleurosigma*, Western Pacific Ocean

## Abstract

A new species of pelagic diatom, *Pleurosigmapacificum***sp. nov.**, is described from the tropical Western Pacific Ocean. It has the typical features of *Pleurosigma*, including a slightly sigmoid raphe, intersected transverse and oblique striae, and loculate areolae with external opening slits and internal poroids. Morphologically, *P.pacificum* belongs to a species group of *Pleurosigma* with lanceolate valves, including *P.atlanticum* Heiden & Kolbe, *P.nubecula* W. Smith, *P.indicum* Simonsen, and *P.simonsenii* Hasle. However, *P.pacificum* differs by its smaller lanceolate valve and smaller intersection angle as well as elliptical areolae without a silica bar. The SSU rDNA and *rbc*L sequence data place *P.pacificum* in a basal position relative to other species of *Pleurosigma*. Our molecular phylogenetic analyses did not support the monophyly of lanceolate and slightly sigmoid species. Thus, the sigmoidality of valve outline cannot be considered as a criterion to define the species group.

## ﻿Introduction

Smith (1852) established the genus *Pleurosigma* W. Smith for some sigmoid naviculoid diatoms. Peragallo (1891) clarified the infrageneric delimitation by combining the orientation of the striae and their angle of the intersection. Species with transverse and oblique striae were separated from those with transverse and longitudinal striae as well as species with centrally interrupted striae. Subsequently, Cleve (1894) transferred all those species with transverse and longitudinal striae into the genus *Gyrosigma* and retained species with transverse and oblique striae in the genus *Pleurosigma.*Hendey (1964) gave a clearer delimitation of *Pleurosigma* and provided chloroplast characters. Round et al. (1990) and Reid (2012) used ultrastructural features for their description of *Pleurosigma*. Currently, *Pleurosigma* is characterized by having two or four ribbon-like plastids, (slightly) sigmoid valves, transverse and two obliquely intersecting striae and loculate areolae with external opeing slits and internal poroids (Round et al. 1990; Reid and Williams 2003; Sterrenburg et al. 2005; Reid 2012). To date, this genus contains more than 700 named taxa (Kociolek et al. 2021).

*Pleurosigma* is a group of diatoms that is widely disturbed in brackish to marine environments (Round et al. 1990; Sterrenburg et al. 2003). It is predominantly found as a large population on sediments (Reid 2012) with a sigmoid valve or raphe, but some *Pleurosigma* species have lanceolate or nearly straight valves and are commonly found in planktonic samples. Metabarcoding data have revealed that *Pleurosigma* species are highly diverse in pelagic ocean (Malviya et al. 2016). However, only five species are known to be pelagic, namely *P.antarcticum* Grunow, *P.atlanticum* Heiden & Kolbe, *P.indicum* Simonsen, *P.simonsenii* Hasle and *P.directum* Grunow (Cleve and Möller 1877–1882; Heiden 1928; Simonsen 1974; Sar et al. 2012).

In this paper, we describe a new species of *Pleurosigma* isolated from the tropical Western Pacific Ocean by using light microscopy (LM) and scanning electron microscopy (SEM). Its phylogenetic position is determined by DNA sequence.

## ﻿Materials and methods

### ﻿Sampling, cultivation and morphological observation

Phytoplankton samples were collected from upper 200 m water column by using a phytoplankton net (64 µm mesh), on the Western Pacific Ocean (7°0.26'N, 141°59.63'E). Single cells of diatoms were isolated using capillary pipettes and cultivated in F/2 medium. Cultures were maintained at 24–26 °C under a light intensity of 120–150 µmol photon/m^2^/s, with a light/dark cycle of 12:12 h. Five milliliters of vegetative cells were fixed with 2.5% glutaraldehyde and then cleaned with hydrogen peroxide (Trobajo and Mann 2019). For LM observation, cleaned samples were mounted on glass slides with Mountmedia (Wako Pure Chemical Industries, Ltd., Osaka, Japan). A Zeiss Imager Z2 microscope (Carl Zeiss Microimaging GmbH, Jena, Germany) with differential interference contrast (DIC) was used for LM observation. The measurement methods of the raphe angle and the intersection angle of the oblique striae followed Sterrenburg (1991). For SEM observations, specimens were placed on coverslips, air-dried and coated with osmium. A Hitachi S-4800 (Hitachi, Ltd., Tokyo, Japan) was used for SEM observation.

### ﻿DNA extraction and sequencing

The DNA was extracted and sequenced according to the method described in Li et al. (2022). Algal cell pellets were obtained by centrifuged 10 mL diatom cultures at 5,000 *g* for 5 min. Total DNA was extracted by using the Plant Genomic DNA Kit (Tiangen Biotech Co., Beijing, China). The small-subunit ribosomal DNA (SSU rDNA), large-subunit ribosomal DNA (LSU rDNA), chloroplast-encoded genes *rbc*L and *psb*C were amplified by polymerase chain reaction (PCR). Forward and reverse strands were amplified using the follow primes (Table 1). The PCR cycles of the four markers follow Alverson et al. (2007). The PCR products were purified using TIANgel Midi Purification Kit (Tiangen Biotech Co., China) and sequenced by Tsingke Biotechnology Co.,Ltd. (Beijing, China).

**Table 1.** Primers used to amplify SSU rDNA, LSU rDNA, *rbc*L and *psb*C fragments for *P.pacificum*.

Name	Marker	Sequence (5′ to 3′)	Reference
*SSU*1	SSU	AACCTGGTTGATCCTGCCAGT	(Medlin et al. 1988)
ITS1DR	SSU	CCTTGTTACGACTTCACCTTCC	(Edgar and Theriot 2004)
D1R	LSU	ACCCGCTGAATTTAAGCATA	(Scholin et al. 1994)
D3Ca	LSU	ACGAACGATTTGCACGTCAG	(Lenaers et al. 1989)
*rbc*L 66+	*rbc*L	TTAAGGAGAAATAAATGTCTCAATCTG	(Alverson et al. 2007)
*rbc*L 1444-	*rbc*L	GCGAAATCAGCTGTATCTGTW G	(Ruck and Theriot 2011)
*psb*C+	*psb*C	CACGACCWGAATGCCACCAAT	(Alverson et al. 2007)
*psb*C-	*psb*C	ACAGGMTTYGCTTGGTGGAGTGG	(Alverson et al. 2007)

### ﻿Molecular phylogenetic analyses

To examine the phylogenetic position of *P.pacificum*, a two-gene dataset (SSU rDNA–*rbc*L) including 30 recognized species and 12 unnamed strains, was used to construct the phylogenetic trees (Suppl. material 1). Due to the lack of LSU rDNA and *psb*C data of most *Pleurosigma* species in GenBank, the two genes were not used for the phylogenetic analysis. Since a previous molecular phylogenetic study indicates that Pleurosigmataceae is closely related to *Haslea* and *Navicula* (Li et al. 2017), we selected all the available sequence of Pleurosigmataceae and *Haslea* species in GenBank for analysis. For *Navicula* species, we selected sequences of species with voucher slides or reliable morphological data.

The SSU rDNA and *rbc*L sequences were aligned using MAFFT v.7.313 (Katoh and Standley 2013) with normal mode and Q-INS-I strategy which considered the secondary structure of RNA, respectively. The trimAl was used to trim the alignment with parameter automated1 (Capella-Gutiérrez et al. 2009). The final concatenated alignment included 2,224 positions, of which 1,535 columns were SSU rDNA and 689 were *rbc*L. PartitionFinder 2 was used to select best-fit models for ML and BI analysis (Lanfear et al. 2017), according to the Bayesian information criterion (BIC). The *rbc*L gene was partitioned by codon position. IQ-TREE v.1.6.8 (Nguyen et al. 2015), Mrbayes v.3.2.7 (Huelsenbeck and Ronquist 2001) and TNT v.1.6 (Goloboff and Morales 2023) were used to perform maximum likelihood (ML), Bayesian inference (BI) and maximum parsimony (MP) analysis, respectively. The ML analysis with 1,000 bootstrap was executed with the default settings. The BI program was run for 10^7^ generations with samples every 1,000 generations and the first 25% of trees were discarded as burn-in. Convergence was judged based on the average standard deviation of split frequencies (all less than 0.01) and the ESS values (more than 3,000) were analyzed in the R package RWTY (Warren et al. 2017). The consensus topology and posterior probabilities of all branches were derived from the remaining trees using a majority-rule consensus approach. In the MP analysis, we used a traditional search with TBR branch swapping on 1,000 replicates and holding 10 trees per replication. The resulting 31 most parsimonious trees (MPTs) were used to calculate a strict consensus tree; Standard bootstrap and Jacknife (with 35 removal probability) analyses were performed using a traditional search and 1,000 replicates, with outputs saved as frequency differences. FigTree v.1.4.4 and Adobe Illustrator were used to view and edit trees.

## ﻿Results

### ﻿ Pleurosigma pacificum sp. nov.

Taxon classificationPlantaeNaviculalesPleurosigmataceae

B2483978-A8C5-5523-A3D7-05CE762E04B3

Fig. 1 , Table 2

#### Description.

**Figure 1.**
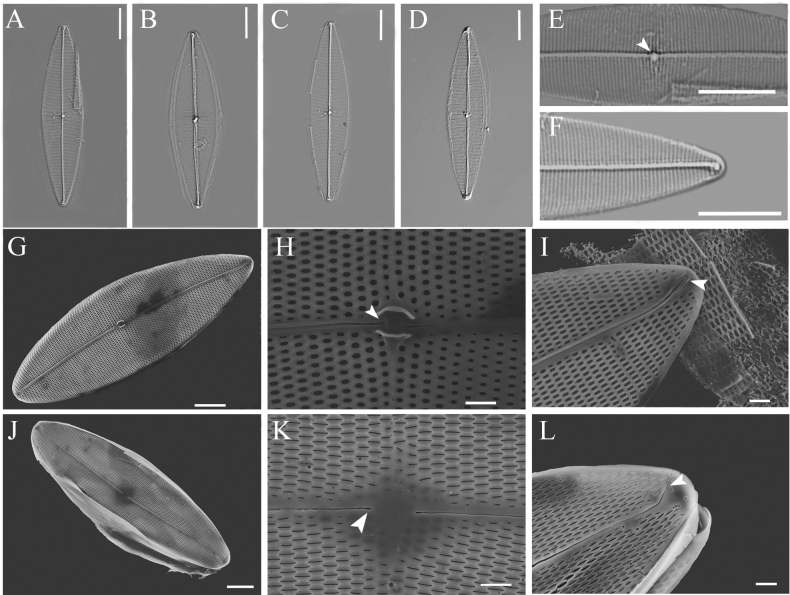
**A–F**LM photographs of *P.pacificum***A–D** cleaned frustules showing parallel transverse striae under LM**E** valve center showing a roundish central area (arrowhead) **F** funnel-shaped terminal area in apical position **G–L**SEM photographs of *P.pacificum***G** internal view of the whole valve **H** internal valve central area showing straight, slight expanded raphe fissures (arrowhead) and nodule bordered by two arched bars **I** internal raphe fissures terminate in helictoglossae at the apices (arrowhead) **J** external view of the whole valve showing the terminal fissures curving into opposite directions **K** external central area of valve showing straight, simple raphe fissures (arrowhead) **L** terminal fissures (arrowhead). Scale bars: 10 μm (**A–F**); 5 μm (**G**, **J**); 1 μm (**H**, **I**, **K**, **L**).

Valves lanceolate, gradually tapering towards the subacute ends, 45.0–51.5 µm long and 13.0–15.6 µm wide (Fig. 1A–D). Raphe filiform, straight, slightly curved near poles (Fig. 1A–D). Raphe angle +2° to +4°. Valve center roundish (Fig. 1E, arrowhead). Terminal area funnel-shaped (Fig. 1F). Transverse striae straight, parallel throughout, 21–22 in 10 µm, oblique striae 21–23 in 10 µm, intersecting at an angle between 32° to 35°.

In SEM, internally, raphe fissures terminate in helictoglossae at apices, orientated in opposite direction to one another (Fig. 1G, I, arrowhead). Internal central fissures straight, slightly expanded (Fig. 1H, arrowhead). Central nodule is raised and flanked by two central bars (Fig. 1H). External central raphe fissures straight, simple (Fig. 1K, arrowhead). Terminal fissures bent to opposite side of valve, not extending onto valve mantle (Fig. 1L, arrowhead). Areolae loculate with external opening slits and internal poroids (Fig. 1G, J).

#### Holotype.

MBMCAS286904, an example is illustrated in Fig. 1A, E, F. This slide was deposited in the Marine Biological Museum, Chinese Academy of Sciences, Qingdao, China.

#### Isotype.

MBMCAS286905, an example is illustrated in Fig. 1C. This slide was deposited in the Marine Biological Museum, Chinese Academy of Sciences, Qingdao, China.

#### Type locality.

7°0.26'N, 141°59.63'E, the upper 200 m water column in the tropical Western Pacific Ocean.

#### Etymology.

Named after the Pacific Ocean where the species was discovered.

#### Distribution and ecology.

*Pleurosigmapacificum* is a planktonic species known only from the type locality. The water temperature was 28.5 °C and salinity about 33.4 during sampling.

#### Gene sequences.

These sequences were deposited in the GenBank (SSU rDNA OQ437519, LSU rDNA OQ549922, *rbc*L OQ473490 and *psb*C OQ437519).

#### PhycoBank registration.

http://phycobank.org/103761.

##### ﻿Molecular phylogenetic analyses

**Figure 2.**
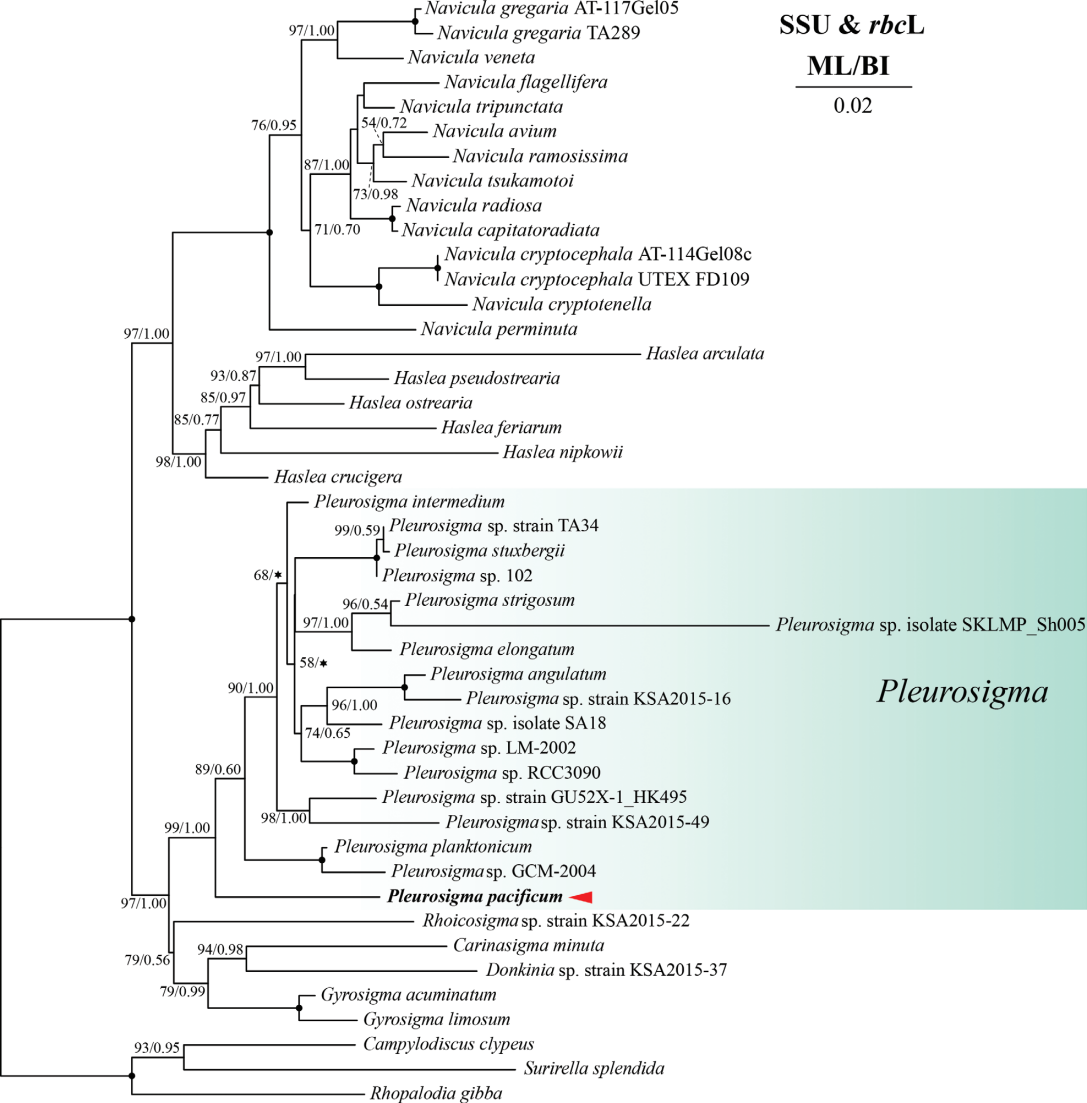
Maximum likelihood (ML) and Bayesian inference (BI) phylogenetic trees based on the concatenated SSU rDNA and *rbc*L sequences. The values on each node indicate ML bootstrap and Bayesian posterior probabilities (%), respectively. Only bootstrap values over 50% are shown on the tree. Black dot indicate ML/BI = 100/1.00. The asterisk indicates the topological incongruence between ML and BI trees.

The BLASTn search showed that the SSU rDNA sequence of *P.pacificum* shares 97.65% identity with an uncultured marine eukaryote (KC771201). The *rbc*L gene sequence of *P.pacificum* shares 92.54% identity with *P.intermedium* (NC_066077). The ML, BI and MP phylogenetic tree based on the concatenated SSU rDNA and *rbc*L gene showed that *P.pacificum* belongs to the clade of the genus *Pleurosigma* with high support (IQ-TREE ultrafast bootstrap value = 99, Mrbayes posterior probability = 1.00, MP standard boostrap value = 77 and MP jacknife value = 85). The *P.pacificum* is basal to all other species of *Pleurosigma*, and branched earlier than the two slightly sigmoid species, *P.planctonicum* and *P.intermedium* (Fig. 2, Suppl. material 2).

## ﻿Discussion

The valves of *Pleurosigmapacificum* sp. nov. possess intersected transverse and oblique striae, opposite curved raphe distal endings, two internal central bars, and loculate areolae with an internal poroids and an external slit opening. These are considered to be the main characters of the genus *Pleurosigma* (Hendey 1964; Reid 2002; Sterrenburg et al. 2005). Molecular data place *P.pacificum* in a basal position relative to other species of *Pleurosigma*.

The morphological data place *P.pacificum* in a group of species, which includes *P.nubecula* W. Smith, *P.indicum* Simonsen, *P.simonsenii* Hasle, and *P.atlanticum* Heiden & Kolbe, with lanceolate valves and a straight raphe (Table 2). Among them, *P.pacificum* can be easily distinguished from *P.simonsenii* by its much smaller valves (45.0–51.5 µm vs. 300–600 µm long). *P.pacificum* differs from *P.indicum* as its internal poroids of areolae are not bisected by a central bar (Fig. 1G in this study vs. figs 41 and 42 in Sar et al. 2012), and from *P.atlanticum* and *P.nubecula* by the smaller stria angle (32–35° in *P.pacificum*, 60° in *P.atlanticum* and 60–61° in *P.nubecula*).

**Table 2.** Comparison of morphological features of *Pleurosigmapacificum* sp. nov. with similar species.

Characteristics	*P.pacificum* sp. nov.	*P.simonsenii* ^#^	*P.indicum*	*P.atlanticum*	*P.nubecula*
Length (µm)	45.0–51.5	300–600	70–90	69–76	82–85
Width (µm)	13.0–15.6	40–75	8–11	13.0–16.5	16
Raphe angle	2–3°	ND	2.5–2.6°	1.2°	1.2–1.9°
Transverse striae in 10 µm	21–22	28–30	23–28	20	25–26
Oblique striae in 10 µm	21–23	30	20–24	20	23–25
Stria angle	32–35°	60°	44–48°	60°	60–61°
Valve outline	lanceolate	Lanceolate, slightly sigmoid	Lanceolate, slightly sigmoid	Lanceolate	Narrowly lanceolate
Raphe	Straight, slightly deflected near the apices	Straight, sigmoid before the ends	Straight, eccentric towards the ends	Straight, slightly deflected at poles	Very slightly deflected
Apices	Subacute	very acute	Subacute	Blunt rounded obtuse	Subacute
Internal view areolae	elliptical without bars	ND	elliptical, bisected by a narrow silica bar	ND	elliptical, bisected by a narrow silica bar
Sources	This study (n = 20)	Simonsen (1974)	Simonsen (1974); Sar et al. (2012)	Heiden and Kolbe (1928)	Sar et al. (2012)

ND = not documented. ^#^Identified as *P.planctonicum* in original description.

The definition of the genus *Pleurosigma* has undergone constant debate and modifications (Cleve 1894; Hendey 1964; Round et al. 1990; Reid 2012). *Pleurosigma* is characterized by its (slightly) sigmoid valve and raphe, two or four ribbon-like chloroplasts, areolae opening to outside by elongate slits and inside by a poroid, areolae arranged in decussate rows, and central internal raphe ending in a central nodule. Reid (2012) indiates that the equal thickening of the raphe sternum on both sides of raphe is a synapomorphic feature of *Pleurosigma* and further revised the generic definition based on morphological phylogenetic analyses. Although Reid (2012) did not include any slightly sigmoid *Pleurosgima* species, recent studies and the present work showed that all these lanceolate and slightly sigmoid *Pleurosigma* species share this synapomorphic feature as well as the other features mentioned above (Sar et al. 2012; Sterrenburg et al. 2015). However, our molecular phylogenetic analyses did not support the monophyly of these lanceolate and slightly sigmoid species (Fig. 2, Suppl. material 2). Therefore, the sigmoidality of valve outline cannot be considered as a criterion to define the species group.

## Supplementary Material

XML Treatment for Pleurosigma pacificum

## ﻿Additional information

### Author ORCIDs

Fei-Chao Du https://orcid.org/0009-0005-0674-5761

Yu-Hang Li https://orcid.org/0000-0002-5546-1774

Kui-Dong Xu https://orcid.org/0000-0002-5186-519X

### Data availability

All of the data that support the findings of this study are available in the main text or Supplementary Information.

## Supplementary materials

Supplementary material 1 GenBank accession of SSU rDNA and *rbc*L gene sequences Data type tables (pdf file) Explanation note GenBank accession of SSU rDNA and *rbc*L gene sequences derived from the species used in the phylogenetic analysis. https://binary.pensoft.net/file/858195This dataset is made available under the Open Database License (http://opendatacommons.org/licenses/odbl/1.0/). The Open Database License (ODbL) is a license agreement intended to allow users to freely share, modify, and use this Dataset while maintaining this same freedom for others, provided that the original source and author(s) are credited.Fei-Chao Du, Yu-Hang Li, Kui-Dong Xu

Supplementary material 2 Maximum parsimony (MP) phylogenetic trees based on the concatenated SSU rDNA and *rbc*L gene sequences Data type figure (pdf file) https://binary.pensoft.net/file/858196This dataset is made available under the Open Database License (http://opendatacommons.org/licenses/odbl/1.0/). The Open Database License (ODbL) is a license agreement intended to allow users to freely share, modify, and use this Dataset while maintaining this same freedom for others, provided that the original source and author(s) are credited.Fei-Chao Du, Yu-Hang Li, Kui-Dong Xu
