# Supplementary material for: ﻿Morphology and molecular phylogeny of Pleurosigmapacificum sp. nov. (Pleurosigmataceae), a new tropical pelagic species from the Western Pacific Ocean
Source: PhytoKeys. 2023 Jun 2;227:99–108. doi: 10.3897/phytokeys.227.103890 (PMC10257139; doi:10.3897/phytokeys.227.103890)
Supplement: Supplementary material 1 — GenBank accession of SSU rDNA and rbcL gene sequences [file phytokeys-227-099_article-103890__-s001.pdf]

Supplements

Table S1. GenBank accession of SSU rDNA and *rbc*L gene sequences derived from the species used in the phylogenetic analysis.

Species	SSU	*rbc*L
Gyrosigma acuminatum	HQ912598	HQ912462
Gyrosigma limosum	KY320348	KY320287
Navicula radiosa	AM501972	AM710438
Navicula gregaria	AM501974	AM710440
Navicula cryptotenella	AM502015	AM710482
Navicula cryptocephala	AM501973	AM710439
Navicula capitatoradiata	AM502012	AM710479
Navicula tripunctata	KT072979	KT072925
Navicula perminuta	KY320360	KY320299
Navicula gregaria	KY320358	KY320297
Navicula flagellifera	KY320357	KY320296
Navicula cryptocephala	HQ912603	HQ912467
Haslea arculata	MN977830	MN977807
Haslea ostrearia	AY485523	HE663064
Haslea crucigera	AY485482	KC015014
Haslea feriarum	KY937693	KY937696
Haslea nipkowii	AY485488	KY320290
Haslea pseudostrearia	KY320350	HE978356
Navicula avium	KY937692	KY937695
Navicula ramosissima	KY320363	KY320302
Navicula tsukamotoi	KY937691	KY937694
Navicula veneta	AM501970	AM710436
Pleurosigma intermedium	AY485489	
Pleurosigma planktonicum	AY485514	
Campylodiscus clypeus	HQ912412	HQ912398
Rhopalodia gibba	HQ912407	HQ912393

Surirella splendida	HQ912415	HQ912401
Pleurosigma sp. strain TA34	KY320349	KY320288
Pleurosigma strigosum	KJ671702	KJ671810
Pleurosigma angulatum	MN382130	MN380173
Pleurosigma stuxbergii		KT943674
Pleurosigma elongatum		HQ685847
Pleurosigma pacificum	OQ437519	OQ473490
Carinasigma minuta	KX981841	KX981812
Rhoicosigma sp. strain KSA2015-22	MH063504	MH064136
Donkinia sp. strain KSA2015-37	MH063463	MH064087
Pleurosigma sp. strain KSA2015-16	MH063498	MH064130
Pleurosigma sp. 102	KJ961710	
Pleurosigma sp. GGM-2004	AY485515	
Pleurosigma sp. isolate SA18	KX981840	KX981822
Pleurosigma sp. LM-2002	AF525664	
Pleurosigma sp. RCC3090	KT861017	
Pleurosigma sp. strain KSA2015-16	MH063498	MH064130
Pleurosigma sp. isolate SKLMP_Sh005	MG914021	
Pleurosigma sp. strain GU52X-1_HK495	MH040328	MH040277
Pleurosigma sp. strain KSA2015-49	MH063496	MH064129
