# Supplementary material for: ﻿Morphology and molecular phylogeny of Pleurosigmapacificum sp. nov. (Pleurosigmataceae), a new tropical pelagic species from the Western Pacific Ocean
Source: PhytoKeys. 2023 Jun 2;227:99–108. doi: 10.3897/phytokeys.227.103890 (PMC10257139; doi:10.3897/phytokeys.227.103890)
Supplement: Supplementary material 2 — Maximum parsimony (MP) phylogenetic trees based on the concatenated SSU rDNA and rbcL gene sequences [file phytokeys-227-099_article-103890__-s002.pdf]

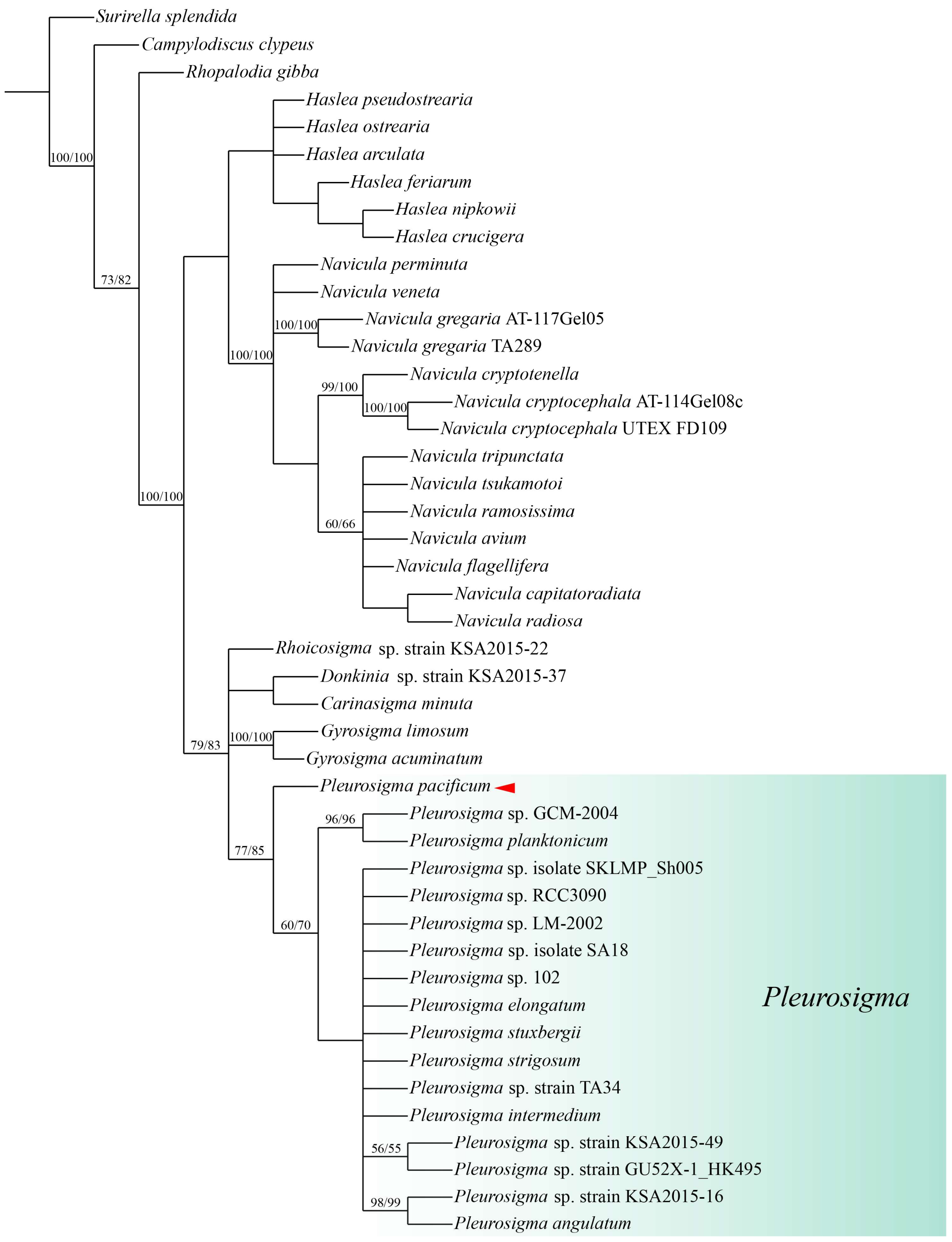

Figure S1. Maximum parsimony (MP) based on the concatenated SSU rDNA and *rbc*L gene sequences. The values on each node indicate MP standard bootstrap and jacknife, respectively. Only standard bootstrap values and jacknife values over 50% are shown on the tree.
